# V-TIME: a treadmill training program augmented by virtual reality to decrease fall risk in older adults: study design of a randomized controlled trial

**DOI:** 10.1186/1471-2377-13-15

**Published:** 2013-02-06

**Authors:** Anat Mirelman, Lynn Rochester, Miriam Reelick, Freek Nieuwhof, Elisa Pelosin, Giovanni Abbruzzese, Kim Dockx, Alice Nieuwboer, Jeffrey M Hausdorff

**Affiliations:** 1 Department of Neurology, Laboratory for Gait Analysis & Neurodynamics, Movement Disorders Unit, Tel Aviv Sourasky Medical Center, 6 Weizmann Street, Tel Aviv 64239, Israel; 2 School of Health Related Professions, Ben Gurion University, Beer Sheba, Israel; 3 Institute for Aging and Health, University of Newcastle, Newcastle, UK; 4 Department of Geriatric Medicine and Neurology, Radboud University Nijmegen Medical Center, Nijmegen, The Netherlands; 5 Department of Neurosciences, Universita Degli Studi Di Genova, Genova, Italy; 6 Department of Rehabilitation Science, Katholieke Universiteit Leuven, Leuven, Belgium; 7 Department of Physical Therapy, Sackler Faculty of Medicine, Tel-Aviv University, Tel-Aviv, Israel; 8 Harvard Medical School, Boston, MA, USA

**Keywords:** Falls, Ageing, Gait, Cognitive function, Prevention, Virtual reality

## Abstract

**Background:**

Recent work has demonstrated that fall risk can be attributed to cognitive as well as motor deficits. Indeed, everyday walking in complex environments utilizes executive function, dual tasking, planning and scanning, all while walking forward. Pilot studies suggest that a multi-modal intervention that combines treadmill training to target motor function and a virtual reality obstacle course to address the cognitive components of fall risk may be used to successfully address the motor-cognitive interactions that are fundamental for fall risk reduction. The proposed randomized controlled trial will evaluate the effects of treadmill training augmented with virtual reality on fall risk.

**Methods/Design:**

Three hundred older adults with a history of falls will be recruited to participate in this study. This will include older adults (n=100), patients with mild cognitive impairment (n=100), and patients with Parkinson’s disease (n=100). These three sub-groups will be recruited in order to evaluate the effects of the intervention in people with a range of motor and cognitive deficits. Subjects will be randomly assigned to the intervention group (treadmill training with virtual reality) or to the active-control group (treadmill training without virtual reality). Each person will participate in a training program set in an outpatient setting 3 times per week for 6 weeks. Assessments will take place before, after, and 1 month and 6 months after the completion of the training. A falls calendar will be kept by each participant for 6 months after completing the training to assess fall incidence (i.e., the number of falls, multiple falls and falls rate). In addition, we will measure gait under usual and dual task conditions, balance, community mobility, health related quality of life, user satisfaction and cognitive function.

**Discussion:**

This randomized controlled trial will demonstrate the extent to which an intervention that combines treadmill training augmented by virtual reality reduces fall risk, improves mobility and enhances cognitive function in a diverse group of older adults. In addition, the comparison to an active control group that undergoes treadmill training without virtual reality will provide evidence as to the added value of addressing motor cognitive interactions as an integrated unit.

**Trial Registration:**

(NIH)–NCT01732653

## Background

Gait impairments and falls are ubiquitous among older adults and patients with common neurological diseases. Approximately 30% of community-dwelling adults over the age of 65 fall at least once a year [1,2]. In persons with Parkinson’s disease (PD), mild cognitive impairment (MCI) or dementia, falls are even more frequent with annual incidence rising to 60–80% [2,3]. The consequences of these falls may be severe, leading to institutionalization, loss of functional independence, disability, fear of falling, depression and social isolation [4].

Most falls occur during walking [5,6] and, not surprisingly, gait impairment has been associated with an increased risk of falls [7,8]. Gait abnormalities in elderly fallers and patients with PD include reduced gait speed, stride length, and increased stride symmetry [9]. Fear of falling, cautious gait [10,11], gait unsteadiness, or dysrhythmicity of stepping have also been recognized as mediators of fall risk [12-15].

There is a growing body of research that specifically links the cognitive sub-domains of attention and executive function (EF) to gait alterations and fall risk [15-21]. EF apparently plays a critical role in the regulation of gait especially under challenging conditions where decisions need to be made in real-time [22]. Walking while avoiding obstacles and walking while simultaneously performing another task, i.e., dual tasking (DT), place greater demands on cognitive resources such as divided attention and executive control, judgment, and reasoning, compared to “single task” walking [23-25]. EF scores and dual tasking gait performance have been associated with fall history and have been shown to predict future falls, even over several years of follow-up [17,21,26]. Although there is no universal agreement, many studies in patients with PD have reported that EF and dual tasking gait abilities are associated with fall risk [27-29] and attention-deficits predict future falls in patients with PD [30]. This may explain why falls occur so frequently among older adults, and even more so in patients with PD and patients with MCI. We suggest that these three groups share cognitive deficits that contribute to and exacerbate their fall risk. MCI patients are cognitively impaired, by definition. As much as 60% of patients who receive the diagnosis of PD already have cognitive deficits [31,32], and many older adults suffer from age-associated decline in cognitive function.

Another risk factor identified as a cause for falls in the elderly is obstacle crossing. Compared to healthy young adults, older adults walk more slowly during obstacle crossing [5,33-36], with smaller steps [34-36] landing dangerously closer to the obstacle with their lead limb [36-38]. Age-related deficits in vision, proprioception and visual-spatial orientation can also negatively impact postural stability and lower limb kinematics when crossing obstacles [5,34,36,37,39]. Obstacle negotiation heavily relies on the availability of ample cognitive resources, due to the need for motor planning and visually dependent gait regulation [40,41].

Many intervention programs based on reported multiple risk factors have been proposed and evaluated to reduce fall risk [42]. However, despite the extensive knowledge on fall risk obtained in recent years, there is no consensus as to the most efficacious or optimal treatment approach [43,44]. Common treatments include exercise programs to improve strength or balance, educational programs, medication optimization, environmental modification and multi-factorial interventions involving a combination of several modalities. To date, however, the effects on fall risk tend to be small and the reported changes are largely focused on motor aspects with limited long-term retention [45-47].

Mahoney [48] suggested that perhaps the reason that multi-factorial interventions are not consistently successful is because they fail to address three major concepts: 1) training should be intensive, focused on the key impairment and become progressively more rigorous; 2) the training should fit the target population; 3) delivery of the intervention should include mechanisms to maximize motor learning and induce a behavioural change. We propose that insufficient focus on cognitive aspects, in particular, the motor-cognitive interactions that contribute to fall risk, might contribute to the sub-optimal success of previous fall risk interventions. Even if cognitive function is targeted, it is generally done so in isolation and the motor-cognitive interactions are not directly addressed in an integrated fashion needed to successfully and safely ambulate in daily living.

**Figure 1:**
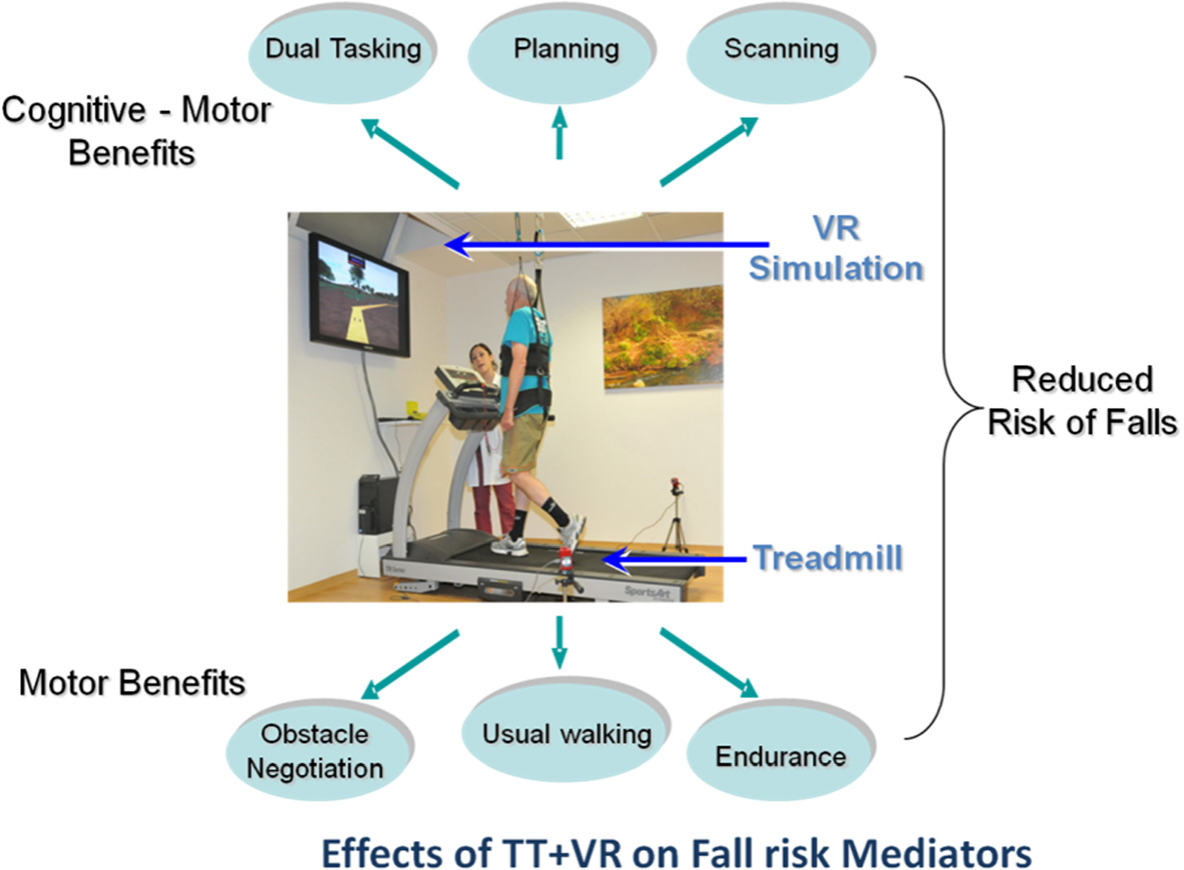
**The V-TIME multi-modal intervention solution for reducing fall risk.** Current treatment of fall risk focuses on motor, e.g., gait, problems. V-TIME focuses on both gait and cognitive deficits to optimally treat multiple, critical fall risk aspects and enhance mobility, physical activity and cognitive function. The current working version of V-TIME is shown. A patient trains on a treadmill while viewing a virtual environment that presents obstacles, different types of challenges, and feedback [49]. Written informed consent was obtained from the patient for publication of this case report and any accompanying images. A copy of the written consent is available for review by the Editor-in-Chief of this journal.

To address this challenge, a multi-modal treadmill training program augmented by virtual reality (VR) (see Figure 1) was developed to deal with both the motor and cognitive aspects of fall risk and to promote motor learning critical for key tasks of safe ambulation. In general, VR is defined as a “high-end-computer interface that involves real time simulation and interactions through multiple sensorial channels” [49-51]. VR can be used to provide training in a more stimulating and enriching environment than traditional rehabilitation whilst providing feedback about performance to assist with learning new motor strategies of movement. Therefore, treadmill training augmented by VR is, theoretically, well-suited as a multi-factorial intervention for fall risk since it is designed to focus on the motor-cognitive aspects of fall risk such as dual tasking, obstacle negotiation and executive function.

In a pilot study [49], 20 patients with PD participated in an intervention based on a VR system for an obstacle navigation task. Patients walked on a treadmill while negotiating obstacles in a VR scene projected on a wall in front of them. They trained for 3 times a week for 6 weeks for about 45 minutes in each session. Visual and auditory feedback was provided by the VR simulation upon error or success and at the end of each walk. After 6 weeks of training, comfortable gait speed significantly improved, as did stride length, gait variability, and over-ground obstacle negotiation. Dual task (DT) performance improved and there was evidence of enhanced task planning and set shifting. Increased gait speeds under all conditions (i.e., comfortable, fast, DT and six minute walk) were not only maintained at follow-up, but also continued to improve 4 weeks later, suggesting that the training generated a positive feedback loop that modified behaviour and overall mobility [49]. Encouraged by these results, an additional pilot study was carried out. Five elderly women who sustained at least 2 falls in the 6 months prior to the study trained using the same treadmill training with VR protocol. Here too, after training, improvements were observed in dual tasking, cognitive function, gait, and mobility, but perhaps the most promising finding was a decrease of 73% in the frequency of falls in the 6 months post-training as compared to 6 months pre-training [52].

The accumulating evidence on the importance of cognitive function to gait and falls combined with these initial findings formed the basis of the present study. The primary aim is to demonstrate that six weeks of treadmill training augmented by VR (TT+VR) reduces the risk of falls in a relatively large and diverse group of older adults (n=300), many of whom will likely have a spectrum of motor and cognitive deficits. The study will compare training effects of TT+VR against an active control paradigm (TT without VR) in a randomized controlled trial. We hypothesize that a 6 week intervention with TT+VR compared to TT alone will reduce the incidence of falls and decrease the risk of falls in elderly adults, patients with PD and individuals with MCI. As a secondary question, we will also explore the neural correlates associated with dual task activation and any plastic effects resulting from the training using imaging techniques. However, protocols for these studies will not be presented in this manuscript.

## Methods/ Design

### Design

**Figure 2:**
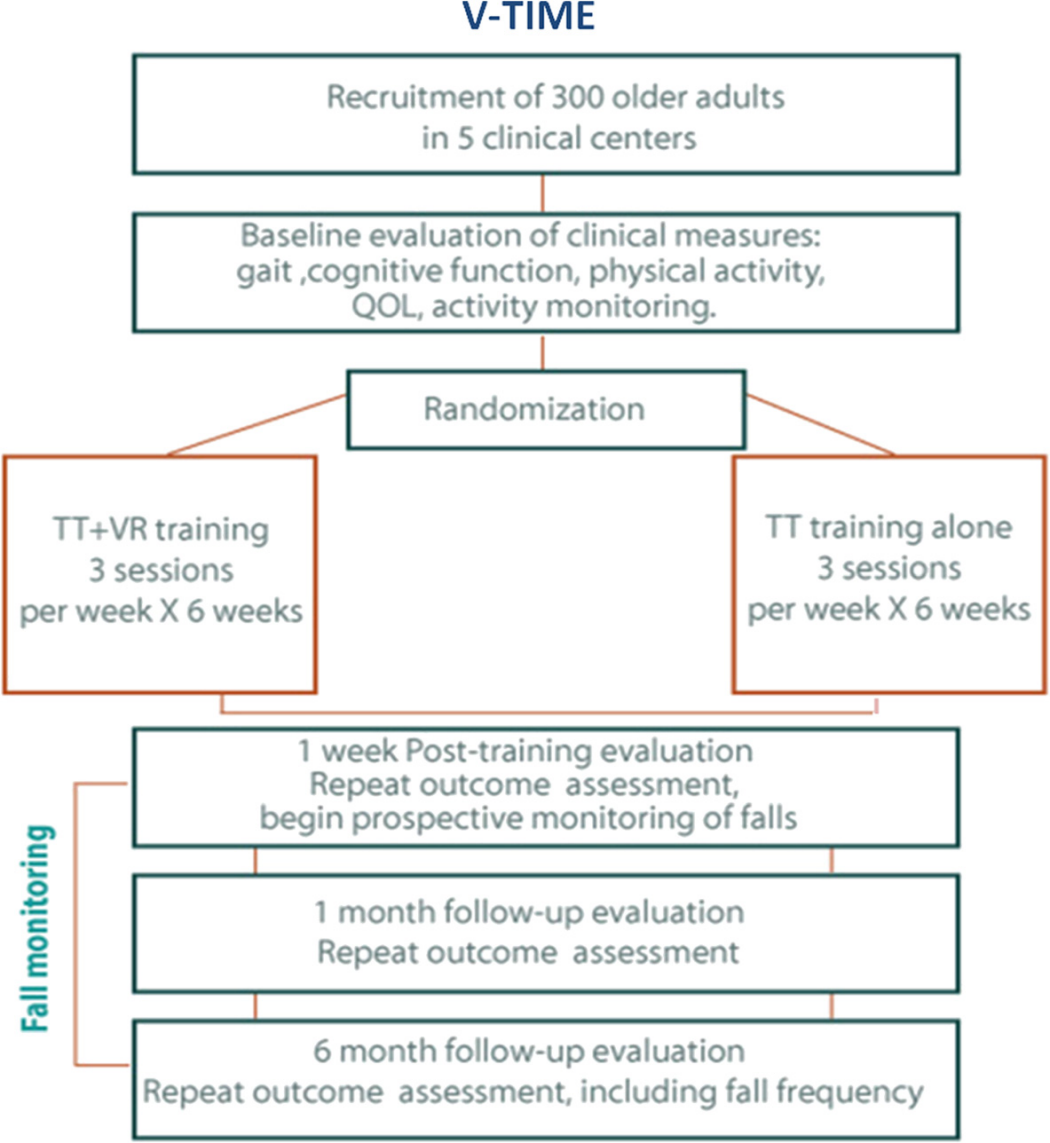
**Summary of the study design and training protocol.** TT: treadmill training. TT+VR: treadmill training augmented by the virtual reality simulation.

A prospective, single blinded, parallel group, randomized controlled trial (RCT) with 6 month follow-up will be employed. The study will include 300 older participants who have experienced two or more falls in the previous 6 months. Participants will be randomized to either the intervention or control group. The intervention group will receive 18 sessions of Treadmill Training with Virtual Reality (TT+VR) and the active control comparison will receive 18 training sessions of treadmill training alone (TT) without the VR simulation (see Figure 2).

### Participants and setting

The participants will be recruited from three groups: older adults with no cognitive impairment (n=100); older adults with mild cognitive impairment (MCI) (n=100) and people with Parkinson’s disease (PD) with no cognitive impairment (n=100). Subjects will be recruited if they meet the following criteria:

#### Common inclusion criteria

• 2 or more falls within 6 months prior to the beginning of the study

• Aged 60–85 years

• Able to walk for 5 minutes unassisted

• Adequate hearing (as evaluated by the whisper test) and vision capabilities (as measured using a Snellen chart).

• Stable medication for the past 1 month and anticipated over a period of 6 months

#### Common exclusion criteria

• Psychiatric co-morbidity (e.g., major depressive disorder as determined by DSM IV criteria)

• Clinical diagnosis of dementia or other severe cognitive impairment (Mini Mental State Exam score <24)

• History of stroke, traumatic brain injury or other neurological disorders (other than PD and MCI, for those groups)

• Acute lower back or lower extremity pain, peripheral neuropathy, rheumatic and orthopaedic diseases

• Unstable medical condition including cardio-vascular instability in the past 6 months

• Unable to comply with the training or currently participating in another interfering therapy or a fall clinics programInterfering therapy

#### Group specific criteria

##### Participants with PD

Inclusion

• Diagnosis of idiopathic PD, as defined by the UK Brain Bank criteria

• Hoehn and Yahr stage II-III

• Taking anti-Parkinsonian medication

• Stable medication for past 1 month and anticipated over next 6 months or stable Deep Brain Stimulation for at least one month and expected following 6 months

Exclusion

• Severe freezing of gait (defined as having >15 on the new FOG questionnaire) [53]

##### MCI group

Inclusion

• Score 0.5 on the Clinical Dementia Rating Scale (CDR)

• Free from any neurological disorders that may have caused the cognitive impairment

### Sample size calculation

The primary outcome measure is fall incidence rates during the 6 month post-training follow-up period. The sample size estimate is based on extrapolations from our pilot studies [49] and other related promising pilot work (e.g., Rosenblat et al. [54], Pai et al. [55] and, Weerdesteyn et al. [56]). Power was set at 80%, alpha was set at 5% and we accounted for drop out rate of 20%. Using a relatively conservative estimate, we assume that the control group fall incidence rate, after intervention, will be three falls per year. If we consider a 40% reduction for the treatment group relative to this, then, during the 6 month monitoring of falls incidence, a total of 166 subjects would be required for 80% power (83 in each group) to detect differences between the two treatment groups assuming non-inferiority with moderate correlations among covariates (R-squared = 0.50). A much smaller sample is needed to detect between group differences for the secondary outcomes. For example, with 22 subjects in the intervention and control arms, we will have 90% power to detect an intervention effect (assuming Cohen’s f for ANOVA of 0.21), in dual tasking gait speed. To enhance our ability to examine the effects of the intervention on fall incidence within the three sub-groups (seniors, PD, MCI), we will aim to recruit 100 subjects per group, for a total of 300 subjects.

### Recruitment and randomization procedure

The study will be conducted in 5 clinical centres across Europe (Lab for Gait & Neurodynamics, Tel Aviv Sourasky Medical Centre, Israel; Department of Rehabilitation Sciences, Katholieke Universiteit Leuven, Belgium; Institute for Ageing and Health, Newcastle University UK; Department of Neurosciences, University of Genoa, Italy; Departments of Geriatric Medicine & Neurology, Radboud University Nijmegen Medical Centre, The Netherlands).

Ethical approval was obtained by ethics committees of each of the above clinical sites. Eligible subjects, who agree to participate, will be asked to provide an informed written consent after which they will be randomized to one of two arms of the study: 1) TT+VR; 2) TT. A permuted blocked randomization procedure will be used selecting randomly from a block size of 4, 6 or 8. Group allocation will be performed by a third party not involved in the day to day running of the study; the treating therapist will be notified by e-mail to ensure concealed allocation.

### Intervention

All interventions will be delivered by therapists trained in the standard protocols across centres in the consortium countries. Consistent with the motor learning literature and the pilot studies [49,52], all subjects will be trained 3 times a week for 6 weeks, each session will last approximately 45 minutes.

### Virtual reality system

Details of the instrumentation are provided elsewhere [49,52]. Briefly, the system includes a camera based motion capture (Kinect) and a computer generated simulation. The camera will be used to collect the movement of the participant’s feet while walking on the treadmill. These images will be transferred into the computer simulation and projected to the patient on a large screen while training, enabling the subjects to see their feet walking within the VR simulation. The virtual environment (VE) will consist of obstacles, different pathways, narrow corridors and distracters, requiring modulations of step amplitude in two planes (i.e., height and width) coordinated with walking behaviour. The speed, orientation, size, frequency of appearance and shape of the targets will be manipulated according to individual needs following a standardized protocol. Environmental features (e.g., visibility, settings and distractions) will be adjusted to increase training complexity. The VE will impose a cognitive load requiring attention and response selection as well as processing of rich visual stimuli involving several perceptual processes. The system will provide visual and auditory feedback of successful or unsuccessful task performance to enhance motor learning. Adaptability of the system is foreseen to adjust training parameters to the clinical needs of the individual participant.

### TT+VR group (the intervention group)

#### Motor aspect of training

Gait speed over-ground will be measured over 10 meters at the beginning of each week of training. This speed will be registered and the treadmill speed will be set accordingly, as detailed below. Training will be divided into bouts of walking and rest breaks in between. The duration of the initial session should be ideally 20 minutes of walking time. A safety harness will be attached to an over head suspension system, but no weight support will be provided.

#### Training progression

Training progression will be based on increasing both motor and cognitive challenges, individualized to the participant’s level of performance. The motor component of training progression will include an increase in the treadmill speed and duration of training. Treadmill speed in the first week will be set at 80% of over ground gait speed. In the second week, the treadmill speed will increase to 90% of over ground speed. Another 10-minutes in duration will be added. From the third week, the speed goal will increase by 10% every week and 1 minute of walking will be added to each of the walking bouts (a total of an additional 3–5 minutes per session compared to the previous session). This progression is subject to the performance and the patient’s ability.

The cognitive components of training progression will include changing the number, size and shape of obstacles, and the frequency, speed and direction at which they appear. The Virtual Environment characteristics will also be manipulated by reducing visibility and adding distracters (e.g., birds, cars). During week 1, obstacles will appear infrequently (e.g., every 30 seconds, at low level of difficulty), be unilateral in direction and the environmental features will be minimal (i.e., high visibility, day time walking, minimal distracters). Based on the subject’s performance in weeks 2 and 3, the frequency of appearance of the obstacles will increase, obstacles will appear on the more challenged side and their features (horizontal vs. vertical) will be individualized. Environmental features will appear with some minimal distracters during weeks 2 and 3. In week 4, subjects will be introduced to a new environment to allow for more diversity in training and to maximize transfer to the real-world. Throughout, training should maintain the ratio of 80:20 success/failure rates in order to enhance motor learning. If subjects are not successful, the difficulty level will be decreased to the level previously achieved and vice versa.

### Treadmill training (TT) (active control) group

The participants will walk on the treadmill without receiving the feedback from the VR. As in the TT+VR group, their gait speed over-ground will be measured at the beginning of each week of training. Progression and the time spent with the trainer will follow the same guidelines as the motor progression of the TT+VR group and will include increasing the duration of each of the walking bouts and increasing walking speed.

### Assessment protocol

A repeated measures design will be employed with assessments performed 1 week pre-training, post-training, and at 1 month and 6 months post intervention (Table 1). A trained assessor in each centre, not involved in training and blinded to group allocation, will perform all assessments. Each participant will be assessed at about the same time of day to avoid variability of performance due to any circadian rhythms or medication intake cycle.

**Table 1:** Assessment of outcome measures across the protocol

	**Category**	**Outcome measures**	**Pre training**	**Post training**	**One month follow up**	**Six month follow up**
Primary outcome measure	Falls	Fall frequency				x
Secondary outcome measures	Gait	Gait speed	x	x	x	x
		Gait variability	x	x	x	x
		2 MWT	x	x	x	x
	Balance and mobility	FSST	x	x	x	x
		SPPB	x	x	x	x
		mini-BEST	x	x	x	x
		Community ambulation	x	x	x	
	Cognitive function	MoCA	x			
		TMT	x	x	x	x
		Mindstreams tests of cogntive function	x	x		x
		Verbal Fluency	x	x	x	x
	Healthy Related Quality of Life	SF-36	x	x	x	x
		FES-I	x	x	x	x
	User satisfaction and views	User satisfaction Questionnaire		x		

2MWT- 2 Minute Walk Test, FSST-Four Square Step Test, SPPB- Short Physical Performance Battery, mini-BEST- The mini-Balance Evaluation Systems Test, MOCA- Montreal Cognitive Assessment, TMT- Trail Making Test, FES-I- Fall Efficacy Scale International.

### Outcome measures

#### Primary outcome measures

The primary outcome measure of the study is fall rate. Participants will keep a falls calendar for 6 months post intervention. Consistent with the recommendations of the Prevention of Falls Network Europe (ProFaNE), a fall will be defined as “an unexpected event in which the participant comes to rest on the ground, floor or lower level”. Each time the participant falls he/she will tick the date on the calendar. These calendars will be returned to the researchers every two weeks in a pre-addressed envelope or using an online electronic calendar. Periodic contact by the research staff with each participant will be used to maximize compliance with the fall calendars.

#### Secondary outcome measures

##### Gait

Gait speed and gait variability under usual and DT conditions and while negotiating physical obstacles will be measured. Participants will be asked to walk in a well-lit corridor under 3 conditions each of 1 minute: i) walking in a comfortable speed, ii) walking while subtracting 3 s from a predefined number (dual task), iii) walking while negotiating two obstacles placed on the floor at specific locations. The GaitRite mat, a sensorized 7 meter carpet (CIR Systems, Inc. Haverton MA), will capture individual footfall data using embedded pressure sensors. This is a valid and reliable method of assessing the spatiotemporal parameters of gait in healthy older adults and in patients with Parkinson’s disease [57]. Spatiotemporal gait characteristics (e.g., gait speed (m/s), stride length (m), stride time (s), swing time (%), asymmetry, and step width (cm)) will be determined. Over-ground obstacle negotiation will be evaluated by placing physical obstacles on the GaitRite. The distance between the heel and the physical obstacle during the loading response of the lead foot will be measured to assess clearance and efficient obstacle negotiation.

Small, lightweight 3 axial accelerometers (APDM, Oregon, USA) will be worn on both feet, both wrists and on the lower back of the participants during all gait measurements to quantify temporal measures such as stride time and gait variability [8]. Gait variability (i.e., the inconsistency from one stride to the next) will be determined by calculating the magnitude of stride-to-stride fluctuations, normalized to each subject’s mean stride time to define the Coefficient of Variation (Coefficient of Variation (CV = (standard deviation/mean) × 100)). Gait variability is a validated and reliable measure reflecting fall risk that has been used with patients with PD [58], older adults [24] and individuals with MCI [59]. Data will be collected at 240 HZ, saved onto a computer and analysed using proprietary software.

Endurance will be assessed using the 2 Minute Walk Test. This performance-based tool was originally developed to assess exercise tolerance among individuals with respiratory disease, but has shown high test retest reliability and validity in assessment of gait endurance in older adults [60] and in individuals with neurological conditions [61,62].

#### Balance and mobility

The Four Square Step Test (FSST) requires subjects to rapidly change direction while stepping forward, backward, and sideway, over a low obstacle. Time to complete the test is measured. The test has been validated in older adults [63] with sensitivity of 85% and specificity of 88-100% in predicting fall risk [63].

The Short Physical Performance Battery (SPPB) consists of three types of physical maneuvers: the balance tests, the gait speed test, and the chair stand test. The SPPB is highly reliable in older adults (ICC=0.83-0.89) and has demonstrated a strong and consistent association with health status measures, in spite of the socioeconomic and cultural differences [64].

The mini-Balance Evaluation Systems Test (mini-BESTest) is a performance based measure differentiating balance problems into 6 underlying systems that may be impaired: biomechanical, stability limits, postural responses, anticipatory postural adjustments, sensory orientation, dynamic balance during gait and cognitive effects. The mini-BESTest has been shown to be a reliable (ICC=0.91) and valid measure of balance in individuals with PD [65].

Community ambulation will be assessed using 1) the Physical Activity Scale for the Elderly (PASE). This 27 item self report questionnaire assess habitual physical activity in the home and community environment. The questionnaire was designed to address cultural differences, is available in multiple languages and has been validated for older adults [66]; 2) a tri-axial accelerometer (Axivity Ltd.) will be worn by the participants for 7 days to quantify and monitor stepping and physical activity. The device which records at 100Hz will be mounted on the trunk (L5) and will derive the following outcome measures: step count, postural transitions, sedentary time, percentage walking time, number and time of walking and sedentary bouts. Data will be obtained one-week pre and post training.

#### Cognitive function

Cognitive function will be assessed using a computerized neuropsychological test battery (Mindstreams®, NeuroTrax Corp., NJ) [67]. The battery assesses different cognitive domains including memory, attemtion, executive function, visual spatial processing and a global cognitive composite. The test battery generates age and education adjusted composite indices of each cognitive domain on an IQ like scale, with the score of 100 representing the estimated population mean normalized for age and education level. The battery has been validated in elderly adults, patients with mild cognitive impairment, and patients with PD and has shown to be useful in predicting falls [17,21,68-70].

In addition, we will also include standardized neuropsychological tests such as the Montreal Cognitive Assessment (MoCA); a rapid screening instrument for global cognitive dysfunction. Different cognitive domains are assessed (attention and concentration, executive functions, memory, language, visuo-constructional skills, conceptual thinking, calculations, and orientation). The MOCA was found to be a valid instrument for cognitive screening in MCI and PD [71,72]. In this study the MoCA will be used as a descriptive measure.

The Trail Making Test (TMT) is a neuropsychological test of visual attention and task switching. It consists of two parts in which the subject is instructed to connect a set of 25 dots as fast as possible while still maintaining accuracy. The test provides information about visual search speed, scanning, speed of processing, mental flexibility, and executive functioning. The TMT is valid and reliable for older adults [73,74] and has been previously associated with decreased gait speed, dual task activity, and obstacle clearance [75].This is a well suited outcome measure given the nature of the training.

Verbal Fluency is a test of working memory and language in which participants have to say as many words as possible from a category in a given time (usually 60 seconds). The test includes both semantic and phonemic sections, is related to executive function, and has been shown to be highly reliable and valid in the elderly population [76].

#### Health-related quality of life

The SF-36 Health Survey is a generic self-report questionnaire designed to address health related quality of life. The SF-36 includes one multi-item scale measuring several constructs such as physical functioning; bodily pain; social functioning; general mental health (psychological distress and psychological wellbeing); vitality (energy/fatigue); and general health perceptions. Criterion validity has been established but the scores could also be divided into two aggregate summary measures; the Physical Component Summary (PCS) and the Mental Component Summary (MCS). SF-36 has been validated for older adults and patients with PD [77,78].

Fear of Falling will be evaluated using the Falls Efficacy Scale-International. The FES-I has been significantly associated with performance-based measures of balance and mobility including gait speed and medial-lateral sway. The scale has been shown to be sensitive to change in older adults with and without cognitive impairments [79,80].

#### User satisfaction and views

A questionnaire was developed by the researchers to assess the satisfaction of the participants from the training and to try and obtain subjective information regarding the usability and efficacy of such an intervention in reducing fear of falling, fall risk and frequency of falls.

### Data analysis

Statistical analysis will be undertaken using SPSS version 19.0 (SPSS Corp, Chicago, IL, USA). All analysis will be conducted on an intention-to-treat principle using all randomized participants. Demographic characteristics and baseline data will be summarized by descriptive statistics using means, standard deviations and 95% confidence intervals for continuous variables, median and inter-quartile ranges for non-normal continuous or ordinal data and percentages for categorical data, and will be evaluated for normalcy and homogeneity. For the primary outcome measure, fall rate will be analyzed by calculating relative risk using negative bionomial regression models that adjust for any potential confounders [81]. Fall rate and fall status (none faller, faller and multiple faller) will then be compared within and between groups. The secondary outcome measures will be analyzed using repeated measures analysis of variance (RMANOVA) to assess differences between groups (intervention) and across time (follow up) for each group of participants and then compared across groups. All data will be adjusted for multiple comparisons.

### Safety considerations and adverse events

All measurements are non-invasive and place the subject at no risk other than those that normally may occur during walking. For some of the patients, in particular those who were not practicing any kind of physical exercise prior to the intervention; there is a slight possibility that subjects might feel some muscle soreness and fatigue after training. To prevent excessive fatigue, subjects will be encouraged to take breaks as needed throughout all study procedures. In addition, the study was designed for gradual increases in intensity which will help to increase endurance and build muscle strength. Virtual reality may cause cyber sickness, a sensation similar to motion sickness. This phenomenon is, however, very rare and is related to highly immersive technology. The proposed study will use a 2D projection to deliver the VR simulation which decreases the risk of developing cyber sickness.

## Discussion

The aim of this study is to establish a practical and feasible solution for enhancing mobility, preventing falls and reducing disability among diverse groups of older adults using a unique intervention that combines treadmill training and virtual reality. What sets this project apart from previous work in this field is that the study simultaneously addresses both motor and cognitive function and their interactions that are key to falls using a large, RCT study design, with an active comparison control for assessing efficacy. Training is provided in a virtual environment that implicitly challenges, teaches, and enhances visual scanning, planning, DT abilities and obstacle negotiation. The additional training goals that aim to enhance the cognitive aspects of mobility have not yet been integrated into common practice and are one of the important added features of the proposed intervention. The unique training program takes onboard all aspects of motor learning in that it probes retention as well as transfer of training to real-world activities to maximize resilient effects.

In a sense, this study also addresses the concepts and concerns raised by Mahoney et al. [48]. The proposed training is intensive, focuses on the key impairment and becomes progressively more rigorous. The training and protocol were designed to meet the needs of a diverse group of older adults including those with cognitive deficits and motor impairment due to neurodegeneration. The intervention maximizes motor learning in order to induce a behavioural change. Moreover, training in the computer-controlled virtual environment makes the therapy and protocol standardized and reproducible.

The training protocol that is at the basis of this study was developed based on recently established guidelines on complex interventions in geriatrics [82-87]. The proposed protocol is based on the needs of the three groups who share a high risk of falls, in part due to cognitive deficits. Focus groups and questionnaires have been used to refine the intervention. Feasibility of using such an intervention was assessed and pilot studies were carried out. Further, the outcome measures are validated and selected to evaluate the effects of the intervention on falls and the motor cognitive interactions that contribute to fall risk. This process enables us to confidently advance into a large randomized controlled trial to explore efficacy in comparison to an active training control group.

Evidence on the efficacy of fall prevention in geriatrics is not yet ideal and large randomized control trials are needed. To promote motor learning required for safe ambulation, fall prevention interventions should include motor and cognitive aspects relating to falls, task-specific and generalized training, with the intervention centred around the user’s needs. The proposed intervention set out to bridge all these needs. The knowledge that will be generated by the results of this study are likely to inform new models of care that combine technology, mobility training, and cognitive remediation to reduce risk of falls and enhance mobility even in a chronic disease profile.

## Conclusions

This randomized controlled trial will demonstrate the extent to which an intervention that combines treadmill training augmented by virtual reality reduces fall risk, improves mobility and enhances cognitive function in a diverse group of older adults. In addition, the comparison to an active control group that undergoes treadmill training without the added virtual reality will provide evidence as to the added value of an intervention that addresses motor cognitive interactions as an integrated unit.

## Competing interests

All authors declare that they have no competing interests.

## Authors’ contributions

AM and JMH participated in designing the study, designing the VR system to be used, writing and reviewing of the manuscript. LR participated in designing the study and writing and reviewing the manuscript. MR, FN and KD participated in reviewing the manuscript, EP, GA and AN participated in designing the study and reviewing the manuscript. All authors read and approved the final manuscript.

## Pre-publication history

The pre-publication history for this paper can be accessed here:

http://www.biomedcentral.com/1471-2377/13/15/prepub
